# Jejunal intussusception due to an atypical lipomatous tumor: a rare case report

**DOI:** 10.1093/jscr/rjab624

**Published:** 2022-02-08

**Authors:** Anastasia Karampa, Christos K Stefanou, Stefanos K Stefanou, Kostas Tepelenis, Periklis Tsoumanis, Konstantina M Ntalapa, Paraskeui Giannouli, George Pappas-Gogos, Konstantinos Vlachos

**Affiliations:** Department of Surgery, University Hospital of Ioannina, Ioannina, Greece; Department of Surgery, General Hospital of Filiates, Filiates, Greece; Department of Surgery, General Hospital of Ioannina, “G. Xatzikosta”, Ioannina, Greece; Department of Surgery, University Hospital of Ioannina, Ioannina, Greece; Department of Ophthalmology, University Hospital of Ioannina, Ioannina, Greece; Department of Nursing, University of Ioannina, Ioannina, Greece; Department of Radiology, General Hospital of Ioannina, “G. Xatzikosta”, Ioannina, Greece; Department of Surgery, University Hospital of Ioannina, Ioannina, Greece; Department of Surgery, University Hospital of Ioannina, Ioannina, Greece

## Abstract

Intussusception in adults is rare, and the clinical symptoms of intussusception are subtle, making the diagnosis quite challenging. Gastrointestinal lipomas are rare benign tumors and are essentially adipose growths, most frequently found within the small intestine wall or mesentery. Limited up-to-date evidence exists regarding such lipomas. Intussusception due to a gastrointestinal lipoma constitutes an infrequent clinical entity, and the diagnosis of duodenal lipoma mainly depends on endoscopy examination, supplemented by computed tomography and magnetic resonance imaging. The present report describes a case of jejunal intussusception in an adult with a history of intermittent colicky abdominal pain located in the left upper quadrant over the last month. Contrast-enhanced computed tomography of the abdomen showed the typical target sign of a small intestinal intussusception along the left upper quadrant and a well-defined, low-density tumor in the intussusception. Exploratory laparotomy revealed jejuno-jejunal intussusception secondary to a lipoma, which was successfully treated with segmental intestinal resection.

## INTRODUCTION

Intussusception is defined as the prolapse of one bowel segment (intussusceptum) into an adjacent part (intussuscipiens; [1, 2]). The incidence of adult intussusception stands at 2–3 cases/million/year and is responsible for ~1–2% of all adult intestinal obstructions [1, 3].

Intussusception is usually idiopathic in children, whereas in adults is secondary due to a leading point [3]. Structural lesions are responsible for most of the cases in adults, with only a minority being idiopathic. Moreover, the possibility of both benign and malignant neoplasia is the leading cause of intestinal intussusception in adults, mandating surgical exploration [2].

Tumors of the gastrointestinal tract originate from the small bowel in 1–2% of all cases, with most of these tumors being benign [4]. Lipomas of the small bowel are the third most common benign tumor, constituting rare adipose growths found within the wall or mesentery of the small intestine. The incidence of intestinal lipomas ranges from 0.035 to 4.4% [2, 5]. Lipomas can arise throughout the gastrointestinal tract, with the small bowel accounting for 25%, and most commonly, they are encountered incidentally since they are usually asymptomatic [1]. However, obstruction, perforation, intussusception, pain and bleeding can complicate their presence. Therefore, it is recommended that these tumors should be removed when discovered. Clinical examination is often inconclusive; thus, many patients are discharged with the diagnosis of irritable bowel syndrome, while at the same time, in almost 50% of cases, the diagnosis of intussusception is made during operation [6]. We report a case of an adult patient who presented to our hospital’s emergency department with colicky abdominal pain. The causative lesion proved to be a jejuno-jejunal intussusception due to an atypical lipomatous tumor. A review of the literature is also performed regarding this rare entity. This case report has been reported in line with the SCARE criteria [7].

## CASE REPORT

A 52-year-old male visited the emergency department with a 1-day history of abdominal pain localized in the left upper quadrant. The pain was described as colicky, and it was associated with vomiting. There was no history of fever, chills and diarrhea. The patient gave a history of intermittent colicky pain in the left upper quadrant over the last month. All the vital parameters were within normal limits.

**Figure 1:**
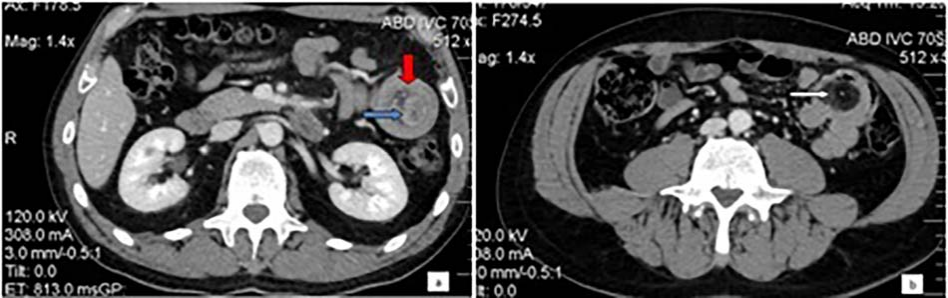
Computed tomography of the abdomen: Bowel-within-bowel configuration in which the layers of the bowel are duplicated forming two concentric enhancing rings by the inner bowel (blue arrow) and the folded edge of the outer bowel (red arrow). Invaginated mesenteric fat and vessels are also visible (**a**). Even further distally within the jejunum the lead point is visualized as an intraluminal mass with fat attenuation, compatible with lipoma (arrow) (**b**).

Physical examination disclosed tenderness in the left upper quadrant without rebound tenderness or muscle guarding. Biochemical and hematological investigations were unremarkable. A contrast-enhanced computed tomography of the abdomen was performed and showed the typical target sign of a small intestinal intussusception along the left upper quadrant and a well-defined, low-density tumor in the intussusception (Fig. 1).

The patient underwent an exploratory laparotomy. A jejuno-jejunal intussusception 10-cm distal to the ligament of Treitz was identified. The involved bowel was reduced by milking the proximal portion while retracting the distal portion without complication. The leading point of intussusception was a 4 cm × 2.5 cm × 2.5 cm palpable soft intraluminal lesion. Resection of the involved segment of the bowel with a 5-cm margin on either side and end-to-end hand-sewn jejuno-jejunal anastomosis was performed. The patient recovered uneventfully, and he was discharged on the eighth postoperative day. Pathological examination confirmed an atypical lipomatous tumor in the submucosal layer of the jejunum.

## DISCUSSION

Intussusception in adulthood is unusual, with an incidence of ~2–3 cases per 1 000 000 individuals per year [4]. Although childhood intussusception is idiopathic in 90% of cases, a mechanical motive and, more specifically, neoplasms are the most frequent cause of adult intussusception [8]. The site of the intussusception is associated with the possibility of malignancy, with colo-colonic intussusception reported having neoplasia as the lead point in 69% of cases and malignancy in 70% [9].

In the small bowel, neoplasia is also the leading cause (57%), but malignancy is less frequent (30%). Adenocarcinoma is the most common malignant lesion in the colon, whereas metastatic melanoma accounts for most of the malignant causes in the small bowel [1]. Rarely, adenocarcinoma, lymphoma and leiomyosarcoma have been reported to be the leading point of small bowel intussusception [2].

The most common benign lesions acting as lead points of intussusceptions are polyps, adenomatous in colon or Peutz-Jeghers in the small bowel and lipomas. Gastrointestinal lipomas are most frequently found in patients aged 50–70 years [1, 10]. Lipomas of the gastrointestinal tract are encountered with decreasing order of frequency in the colon (65–75%), small intestine (20–25%), stomach and esophagus [11]. Their usual location in the small intestine is the ileum (50%), while jejunum is the least common. Lipomas are usually asymptomatic since lesions <1 cm are considered incapable of producing symptoms. However, 75% of those greater than 4 cm are symptomatic due to intussusception, obstruction and hemorrhage [12]. The presenting symptoms and signs are commonly non-diagnostic, and according to the literature, only 32–50% of cases are diagnosed preoperatively.

Imaging studies aid the diagnostic process, with computed tomography (CT) being the imaging method of choice for diagnosing intussusception [12]. The sensitivity of CT scan to diagnose intussusception has been reported from 71.4 to 87.5%, whereas its specificity in adults has been reported to be 100% [11]. When intussusception is suspected, a correct diagnostic algorithm should be to obtain an abdominal ultrasound or a CT scan, since the last is more appropriate in colonic intussusception because of a higher possibility of malignant disease [1].

Regarding treatment options, resection is the treatment of choice for adults presenting with symptomatic intussusception [1, 2, 9, 10]. Partial small bowel resection can either be done by laparotomy or laparoscopy. Laparoscopy can be used if pre-operative imaging shows a benign etiology. The conversion rate from laparoscopy to an open procedure for the treatment of small bowel obstruction ranges from 0 to 16.7% [5]. Discussion continues as to whether or not reduction has to be done before resection. Due to the high percentages of malignant causes of colonic intussusceptions, en-bloc resection seems to be the best option because of the lower risk of perforation or spillage. On the other hand, reduction followed by an elective resection can be defended in cases of small bowel intussusception [3, 5].
